# A Validated Method without Derivatization for the Determination of Gabapentin in Bulk, Pharmaceutical Formulation and Human Urine Samples

**Published:** 2009-06

**Authors:** Rajinder Singh Gujral, Sk Manirul Haque

**Affiliations:** *Vardhman Chemtech Ltd, Nimbua, Dera Bassi, Mohali, Punjab, India*

**Keywords:** gabapentin, high performance liquid chromatography, pharmaceutical formulation, validation

## Abstract

A rapid, sensitive and accurate high performance liquid chromatography with UV detection method was developed and validated for the quantification of gabapentin in bulk, pharmaceutical formulation and human urine samples. Most of the published methods for analysis of gabapentin used derivatization with reagent. The present paper however describes the analysis of gabapentin without any derivatization. The chromatographic separation was carried out on a Waters C_18_ 5 μm column (150 mm × 4.6 mm) using a mixture of methanol – acetonitrile - potassium dihydrogen orthophosphate (pH5.2; 0.028 M) (25:10:65) as a mobile phase with UV detection at 210 nm. The method was linear over the range of 0.1–3.8 mg/ml of gabapentin. The within day and between day precision values are very good.

## INTRODUCTION

**Figure 1:**
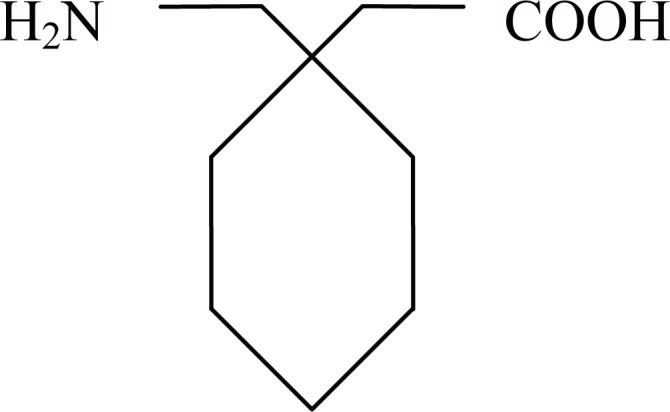
Structure of Gabapentin.

Gabapentin, 1-(amino methyl)-cyclohexane acetic acid (Fig. 1), which is a structural analogue of the inhibitory neurotransmitter γ-amino butyric acid (GABA). It is a potent antiepileptic drug. It is used for the treatment of complex partial seizures, with or without secondary generalization in patient overs 12 years of age (1).The mechanism of action of gabapentin is not completely understood. Recently, it has been shown that the action of gabapentin is possibly due to its high binding to α_2_ – δ protein, an auxiliary submit of voltage – gated calcium channels. Potent binding at this site reduces the release of several neurotransmitters and seizures (2).

There is a wide individual variation in the rate of clearance of this drug and there is always a need to perform compliance testing, ascertain toxicity, and elucidate possible clinical interactions. Accordingly, the therapeutic monitoring of gabapentin is highly desirable (3).

Numerous analytical methods have been reported in the scientific literature for the determination of gabapentin in pure form and pharmaceutical formulation. These methods are based on gas chromatography (4, 5), gas chromatography – mass spectrophotometry (6), high performance liquid chromatography (7–15), capillary electrophoresis (16, 17) and fluorimetry (18–20). But gabapentin has no significant work in ultraviolet and visible absorption spectrophotometry.

Several HPLC methods for determination of gabapentin have been published using different derivatizing agent reagents such as potassium iodate and potassium iodide (7), phenylisothiocyanate (9), 4 – chloro- 7-nitrobenzofurazon (13), 9 – fluorenyl methyl chloroformate (14) and 1, 2 – naphthoquinone – 4 – sulphonic acid sodium salt (15) Most of the HPLC assay procedures for the determination of gabapentin are based on the same approach, involving a simple automated O-opthaldehyde (OPA) (10, 11) derivatization followed by HPLC separation in acidic mobile phases and fluorimetric detection. These methods could be optimized for the determination of gabapentin in dosage forms but they suffer from some limitations such as a lengthy run time or using special reaction conditions which may not be suitable for routine analysis. Using OPA as derivating agent, the fluorescent OPA – drug derivative should be injected immediately after preparation because of the instability of the adduct. Therefore, the method is difficult to apply for routine studies especially when automated instrumentation is not available. Derivatization with potassium iodate and potassium iodide (7) requires more reagents and it detected at higher UV wavelength but it is a good method for anlysis. Derivatization with phenyl - isothiocyanate (PITC) is simple, but this reagent degrades in contact with water and the reaction medium should be completely free from water before the addition of reagent (9) Using 4 – chloro – 7 nitrobenzofurazon as a labeling reagent, the adduct should be extracted and detected by spectrofluorimetry (13).The reaction time for gabapentin and 9 – fluorenyl methyl chloroformate is not too long (about 10 min), but a column temperature of 60°C is needed for separation of the adduct (14). In the method reported by Sagirli *et al* (15) derivatization with 1, 2 – naphthoquinone – 4 – sulphonic acid and extraction of the reaction product were needed and the total run time of the HPLC method was 15 min.

Currently none of the gabapentin dosage forms are indexed in any pharmacopoeia. A direct HPLC method at 215 nm is reported for analysis of gabapentin in bulk drug in the 2007 USP (21). To the best of our knowledge; a few methods have been reported in the literature for determination of gabapentin in pharmaceutical formulations. In one of the methods, spectrofluorimetric determination of gabapentin was reported after derivatization with fluorescamine (20). In another study, colorimetric determination of gabapentin was studied based on the reaction with vanillin and ninhydrin (22). In these cases, the derivatization condition was time consuming and the stability of the reaction products depends on experimental conditions such as pH, temperature and reaction time.

The aim of the present study was to develop a rapid, sensitive, accurate and precise HPLC method for determination of gabapentin without derivatization in bulk, pharmaceutical formulation and human urine samples.

## EXPERIMENTAL

### Materials

- Gabapentin (Vardhman Chemtech Ltd, Nimbua, Punjab, India) used as internal standard (0.9923 mg/mg), standardized with Standard USP Gabapentin Lot No GOE005 (0.999 mg/mg);
- Potassium dihydrogen orthophosphate (KH_2_PO_4_) was purchased from Qualigens fine chemicals, Mumbai, India;
- Acetonitrile and methanol were HPLC grade purchased from Qualigens fine chemicals, Mumbai, India;
- All other chemicals were of analytical grade and used without any further purification.

### Instrumentation

- A Shimadzu LC – 2010 CHT (LC solution softwere), 710 plus Auto sampler (Shimadzu, Kyoto, Japan);
- Waters 2487 (Breeze softwere) consisted of 515 pumps (Waters, Singapore);
- Digital pH meter, G – 2001 A, HPG systems (Mumbai, India).

### Standard Solutions

- Stock standard solution of gabapentin was prepared by dissolving an appropriate amount of the compound in mobile phase to give a final concentration of 5 mg/ml. Standard solutions of gabapentin (0.75, 2.0 and 3.2 mg/ml) were prepared by subsequent dilution;
- A phosphate buffer containing 0.028 M KH_2_PO_4_ (2.50 gm in 650 ml distilled water) and adjusting the pH to 5.2 by adding 10 % NaOH (10 gm in 100 ml distilled water).

### Chromatographic conditions

Separation was achieved using Waters C_18_ 5 μm column (150 mm × 4.6 mm) (Waters, Singapore). The isocratic mobile phase pumped at a flow rate of 1.0 ml/ min consisted of methanol – acetonitrile – potassium dihydrogen phosphate (pH5.2; 0.028 M) (25:10:65, v/v) prepared daily and degassed by passing through a 0.45 μm Millipore filter and ultrasonication for 10 min. All separations were performed at room temperature with detection at 210 nm.

## METHODS

### Procedure for determination of gabapentin

**Figure 2:**
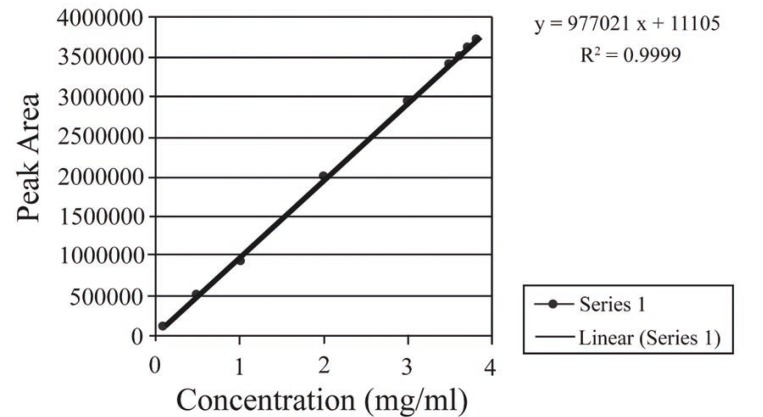
Linear regression plot of the proposed method.

Aliquots of stock solution (5 mg/ml) were transferred in to a 10 ml volumetric flask and volumes were completed to the mark with the mobile phase to produce solutions in the concentration range 0.1–3.8 mg/ml (Fig. 2). Twenty micro liters of the solution was injected into the HPLC system. The eluents were detected by the UV detector with the wavelength of 210 nm. The signals emerging from the detector were integrated as peak area and a calibration graph of peak area against the concentration of gabapentin was plotted. Alternatively, the regression equation was derived.

### Procedure for pharmaceutical formulation

The content of one gabacom tablet was combined and weighed. An amount of powder equivalent to about 800 mg gabapentin was accurately weighed, transferred to a 100 ml volumetric flask, make up to volume with distilled water and placed in an ultrasonic bath for 15 min. After passing through a 0.45 μm Millipore filter, the solution was diluted with water to obtain a concentration of about 8 mg/ml. The drug concentrations of seven replicates were determined by HPLC using the calibration curve.

### Procedure for reference method (21)

Aliquots of stock solution (14 mg/ml) were transferred into a set of 10 ml volumetric flasks and volumes were completed to the mark with diluents (dissolve 2.32 gm monobasic ammonium phosphate in 1000 ml of water. Adjust with phosphoric acid to a pH of 2.0) to produce solutions in the concentration range of 1000-10000 μg/ml. Calibrations were constructed by plotting peak area against the final concentration gabapentin.

For reference method the separation was achieved by using 4.6 mm × 25 cm column that contains packing L1. The isocratic mobile phase pumped at a flow – rate of 1.0 ml/min consisted of acetonitrile – buffer solution [Dissolve 0.58 gm of monobasic ammonium phosphate and 1.83 gm of sodium perchlorate in 1000 ml of water. Adjust with perchloric acid to a pH of 1.8].

### Procedure for the determination of gabapentin in human urine samples

Aliquot volumes of human urine samples were transferred into small separating funnel. 5 ml of carbonate buffer pH – 9.4 (prepared by dissolving 26.5 gm sodium carbonate and 21.0 gm sodium bicarbonate in 500 ml distilled water) was added and solution was mixed well. The solution was then extracted with 3 × 5 ml of diethyl ether. The ether extract was collected and evaporated. The residue was dissolved in 5 ml of distilled water and above general procedure was then followed. The nominal content of gabapentin was determined from the corresponding regression equation.

## METHOD VALIDATION

### Solution stability

The stability of the reference gabapentin and quality control sample solutions at room temperature was evaluated with the help of HPLC systems.

### Specificity and selectivity

The specificity and selectivity of the proposed method was evaluated by estimating the amount of gabapentin in the presence of common excipients such as sodium stearyl fumarate, magnesium stearate, starch, lactose, glucose, fructose and talc.

### Linearity

The linearity of the method was ascertained by taking gabapentin at nine concentration levels 0.1–3.8 mg/ml. Each concentration was independently analyzed five times.

### Accuracy and Precision

The accuracy and precision of the method was evaluated within the linear range based on the analysis of gabapentin in reference standard samples at 0.75, 2.0 and 3.2 mg/ml. Five independent analysis were performed at each concentration level within one day (intra day precision) as well as for five consecutive days (inter day precision).

### Recovery studies

Recovery experiments were carried out by standard addition method. For this, 0.25 (or 0.70, 1.15, 1.75, 2.20, 2.70 and 3.0 mg/ml) of reference gabapentin solution (5 mg/ml) was transferred into a 50 ml volumetric flask followed by 0.25 mg/ml of sample solution (5 mg/ml) and the volume was completed up to the mark with the mobile phase. The total amount was determined by the proposed procedure.

### Results and discussions

**Figure 3:**
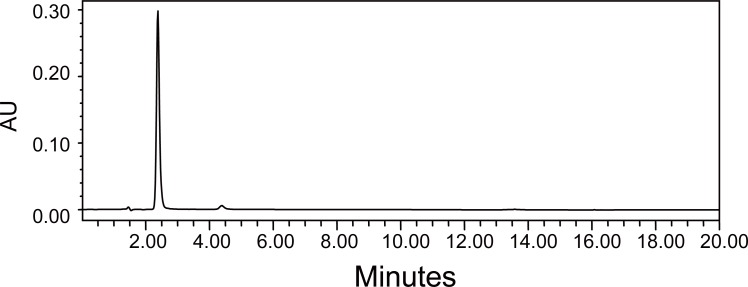
HPLC Chromatograms of Gabapentin (2 mg/ml).

Optimization of chromatographic conditions was achieved by monitoring varying columns and mobile systems. Silica columns such as a μ Bondapak column with different mobile phases did not give a suitable peak shape for analysis. On the other hand, Waters C_18_ column gave better results. After trying different ratios of mixtures of methanol – acetonitrile – potassium dihydrogen orthophosphate (pH5.2; 0.028 M) (25:10: 65, v/v) as mobile phase. Excellent chromatographic specificity with no interference from dosage form excipients was observed. Moreover, a suitable retention time for gabapentin was achieved. Typical chromatograms obtained from the standard solution of gabapentin, assay preparation of Gabacom tablet and a test solution from dissolution medium of Gabacom tablet are presented. Under the chromatographic conditions described, gabapentin was well resolved and eluted at about 2.36 min (Fig. 3); the total run time was within 30 min. Good baseline resolution and peak shape can be observed.

### Solution stability

The solution stability was ascertained from HPLC peak area of reference standard and quality control samples. The peak area was obtained at 2.36 min retention time with a UV detector of wave length of 210 nm (2.000 AUFS). The standard and quality control sample solutions were kept at bellow 5°C for 15 days, it was observed that there was no change in peak area of these solutions.

### Robustness

The robustness of the method relative to each operational parameter was checked and investigated. The operational parameters were also follows:

- Mobile phase, ± 2.5 %
- Buffer pH, 5.2 ± 0.01 %
- Retention time, 20 ± 2 Min

The robustness of the method was assessed by analyzing the active gabapentin in Gabacom tablet. The quality control sample solution containing 2.87 mg/ml of the drug assayed. The percent recovery ± RSD of the method (100.12 ± 0.919) were found to be appreciable, indicating that the proposed method is robust.

### Ruggedness

For the evaluation of ruggedness of the proposed method, the contents of gabapentin at 3.5 mg/ml were assayed following the recommended procedure using Shimadzu LC 2010 CHT Auto sampler and Waters 2487 HPLC systems. The recoveries ± RSD resulting from the Shimadzu LC 2010 CHT (99.86 ± 0.669) and Waters 2487 (99.16 ± 0.471) were compared.

### Accuracy and Precision

Under the optimum experimental conditions, the peak area – concentration plot for proposed method was found to be rectilinear over the range of 0.1 – 3.8 mg/ml. Linear regression analysis of calibration data gave the regression equations cited in Table 1 with correlation coefficients close to unity.

**Table 1:** Optical characteristics and statistical data of the regression equation for the proposed method

Parameters	Proposed Method

Beer’s law limit (mg/ml)	0.1 – 3.8
Linear regression equation	P = 977021 X + 11105
Slope	977021
Intercept	11105
Correlation coefficient	0.9999

The within day precision assays were carried out through replicate analysis (n=5) of gabapentin corresponding to 0.75, 2.0 and 3.2 mg/ ml for the proposed method. The interday precision was also evaluated through replicate analysis of the pure drug samples for five consecutive days at the same concentration levels as used in the within day precision. The results of these assays are reported in Table 2. As can seen from Table 2 that RSD values for within day precision was lower than 0.116 %; RSD values for interday precision was lower than 0.487 %. The precision results are satisfactory.

**Table 2:** Test of precision of the proposed method

Proposed Method	Concentration (mg/ml)		SD	RSD%	SAE	CL
	Taken	Found				

Intra day Assay	0.75	0.735	0.001	0.116	0.038	0.106
	2.00	1.988	0.009	0.471	0.419	1.162
	3.20	3.213	0.009	0.284	0.408	1.131
Inter day Assay	0.75	0.731	0.007	1.009	0.330	0.916
	2.00	1.987	0.019	0.979	0.870	2.414
	3.20	3.213	0.016	0.487	0.699	1.941

The accuracy of the proposed method was ascertained by recovery studies using the standard addition method. The results are summarized in Table 3. The RSD of the proposed method was lower than 0.116 ± 1.884 %. The results agreed well within the acceptable limits of ± 2%. The selectivity of the proposed method was ascertained by analyzing standard gabapentin in the presence of excipients such as sodium stearyl fumarate, magnesium stearate, starch, lactose and talc. It was observed that these excipients did not interfere with the proposed method.

**Table 3:** Determination of gabapentin in pharmaceutical preparation by standard addition method

Formulation	Concentration (mg/ml)			RSD%	SAE	CL
	Taken	Added	Found			

Gabacom	0.25	0.25	0.492	0.181	0.040	0.110
	0.25	0.70	0.965	0.116	0.050	0.139
	0.25	1.15	1.415	1.709	1.081	3.002
	0.25	1.75	2.015	1.884	1.697	4.711
	0.25	2.20	2.451	0.935	1.025	2.846
	0.25	2.70	2.958	0.574	0.008	0.022
	0.25	3.00	3.471	0.471	0.731	2.029

The proposed method was applied to the determination of gabapentin in its pharmaceutical formulation. The result of the proposed method was compared with those obtained by the reference method (21) (Table 4).

**Table 4:** Determination of gabapentin by proposed method and reference method (21)

GabapentinPurity (%)	Proposed Method					Reference Method				
	Taken (mg/ml)	Found	RSD%	SAE	CL	Taken (mg/ml)	Found	RSD%	SAE	CL

99.23	3.5	3.471	0.471	0.007	0.022	3.5	3.485	0.587	0.009	0.025
94.56	3.5	3.293	0.785	0.012	0.032	3.5	3.272	1.323	0.019	0.053
88.65	3.5	3.175	1.565	0.022	0.062	3.5	3.128	1.408	0.020	0.055

The performance of the proposed method was studied with other existing HPLC method (8, 12). In this case, the accuracy and RSD values of the proposed method is better as compared to reported methods, and also the drawback of reported methods (8, 12) are that it requires complex system for dissolution of samples, dissolution medium for the analysis and the method is not applied for in vitro determination of gabapentin in human urine samples.

The proposed method also validated with low purity (94.56% and 88.65%) gabapentin with reference method (21) (Table 4). Therefore, the proposed method is simple and can compete with other existing HPLC method for the determination of gabapentin.

The proposed method was further extended to the *in vitro* determination of gabapentin in human urine samples in the proposed linearity range. The results of analysis are summarized in Table 5. These results are satisfactorily accurate and precise. Therefore the proposed method was found to be accurate for *in vitro* determination of gabapentin in human urine samples.

**Table 5:** Application of the proposed HPLC method to the determination of gabapentin in human urine samples

Amount added (mg/ml)	Amount found (mg/ml)	Recovery (%)

0.20	0.1991	99.55
0.50	0.4989	99.78
1.00	0.9993	99.93
1.50	1.4994	99.96
2.00	1.9991	99.95
2.50	2.4983	99.93
3.00	2.9695	98.98
3.50	3.4813	99.47
X		99.69
RSD		0.347

### Application

The proposed procedure has been successfully applied to quantitative determination of gabapentin in pharmaceutical formulation. The assay of the same batch of samples was also completed by reference method. The results of the proposed method were compared with those obtained by reference method in terms of RSD, SAE and CL values (Table 4). As can be seen from Table 4, the assay results showed good agreement between the proposed method and the reference method. This proposed method is also applicable for the determination of low purity (94.56 % and 88.65%) gabapentin (Table 4).

### Conclusion

The proposed method does not require any laborious clean up procedure before measurement. In addition, the method has wider linear dynamic range with good accuracy and precision. The method shows no interference from common excipients. The statistical parameter and recovery data reveal the good accuracy and precision of the proposed method. Therefore, it is concluded that the proposed method is simple, sensitive and rapid for determination of gabapentin in bulk, pharmaceutical formulation and human urine samples.
